# Gene Targeting Without DSB Induction Is Inefficient in Barley

**DOI:** 10.3389/fpls.2016.01973

**Published:** 2017-01-05

**Authors:** Mihaly Horvath, Hans-Henning Steinbiss, Bernd Reiss

**Affiliations:** Plant DNA Recombination Group, Max Planck Institute for Plant Breeding ResearchCologne, Germany

**Keywords:** gene targeting, gene replacement, genome complexity, acetolactate synthase, barley, *Hordeum vulgare*, positive negative selection, repetitive DNA

## Abstract

Double strand-break (DSB) induction allowed efficient gene targeting in barley (*Hordeum vulgare*), but little is known about efficiencies in its absence. To obtain such data, an assay system based on the *acetolactate synthase* (*ALS*) gene was established, a target gene which had been used previously in rice and *Arabidopsis thaliana.* Expression of recombinases RAD51 and RAD54 had been shown to improve gene targeting in *A. thaliana* and positive-negative (P-N) selection allows the routine production of targeted mutants without DSB induction in rice. We implemented these approaches in barley and analysed gene targeting with the ALS gene in wild type and RAD51 and RAD54 transgenic lines. In addition, P-N selection was tested. In contrast to the high gene targeting efficiencies obtained in the absence of DSB induction in *A. thaliana* or rice, not one single gene targeting event was obtained in barley. These data suggest that gene targeting efficiencies are very low in barley and can substantially differ between different plants, even at the same target locus. They also suggest that the amount of labour and time would become unreasonably high to use these methods as a tool in routine applications. This is particularly true since DSB induction offers efficient alternatives. Barley, unlike rice and *A. thaliana* has a large, complex genome, suggesting that genome size or complexity could be the reason for the low efficiencies. We discuss to what extent transformation methods, genome size or genome complexity could contribute to the striking differences in the gene targeting efficiencies between barley, rice and *A. thaliana*.

## Introduction

Barley (*Hordeum vulgare*) is a commercially important crop and a diploid model cereal for more complex Triticeae, like hexaploid wheat (Mayer et al., 2011). In addition, extended mutant collections and TILLING populations (Lundqvist and Franckowiak, 2003; Gottwald et al., 2009; Lundqvist, 2014) exist and the barley genome is sequenced (International Barley Genome Sequencing Consortium et al., 2012). In particular, the genome sequence enables the use of reverse genetics tools like genome editing and gene targeting. Both technologies are not well developed in barley. Genome editing relies on double-strand break (DSB) induction and subsequent repair by non-homologous end-joining (NHEJ). Repair by NHEJ is often imprecise thereby introducing small deletions and/insertions around the DSB in the target gene (Voytas, 2013; Osakabe and Osakabe, 2014; Puchta and Fauser, 2014). Genome editing allows targeted mutagenesis with high efficiency in plants, including barley (Wendt et al., 2013; Gurushidze et al., 2014). In gene targeting or gene replacement, homologous recombination (HR) is the driving force for homology-mediated DNA integration. In addition, gene targeting had been possible long time before genome editing became available and works also in the absence of DSB induction. However, the efficiencies obtained with induced DSBs are consistently higher in plants (Reiss, 2003; Puchta and Fauser, 2013), including barley (Budhagatapalli et al., 2015; Watanabe et al., 2016). Using induction of a DSB at a transgenic model locus we obtained several events with a limited amount of time and labor before (Watanabe et al., 2016). However, comparable data on gene targeting efficiencies in the absence of DSB induction, or with an endogenous target gene are still lacking.

Gene targeting is a well-established technology in rice (*Oryza sativa*), another important monocot (Iida and Terada, 2004; Endo et al., 2007; Terada et al., 2007; Nishizawa-Yokoi et al., 2015; Saika et al., 2015; Shimatani et al., 2015). Originally, an efficient transformation system using amplified embryogenic callus and positive-negative (P-N) selection had enabled gene targeting long before artificial nucleases became generally available (Terada et al., 2002). Later, a transformation method using scutella (Toki et al., 2006) was developed and a gene targeting assay system built upon the rice *acetolactate synthase* (*ALS*) gene was established (Endo et al., 2007). *ALS* is an endogenous gene encoding an enzyme for the biosynthesis of branched-chain amino acids which is the target of various agronomically important herbicides. A number of different mutations are known that confer herbicide resistance, including one in barley (Lee et al., 2011). *ALS* was widely used to analyze gene targeting in plants including tobacco (*Nicotiana tabacum*) (Lee et al., 1990; Townsend et al., 2009), *Arabidopsis thaliana* (Badur and Reiss, 2004; Prinzenberg, 2006) and rice. Particularly high gene targeting efficiencies with more than 4% of transformed embryos were achieved in rice without any need for DSB induction (Endo et al., 2007) using *ALS*, an Imazethapyr herbicide and a common resistance mutation, suggesting that this plant has a naturally high competence for gene targeting.

To allow a direct comparison of the results with *A. thaliana* and rice, an assay system based on *ALS* was developed in barley which uses the same resistance mutation and herbicide as in *A. thaliana* and rice before. As a prerequisite, the *ALS* gene of the transformable cultivar Golden Promise was isolated, a mutation conferring resistance to the Imazethapyr herbicide Pursuit introduced and the barley transformation and Pursuit selection conditions optimized.

Overexpression of recombination enzymes like the yeast (*Saccharomyces cerevisiae*) ScRAD54 (Shaked et al., 2005; Even-Faitelson et al., 2011) and the *Physcomitrella patens* PpRAD51B protein (Prinzenberg, 2006) were shown to stimulate gene targeting in *A. thaliana*. In addition, P-N selection (Terada et al., 2002) was a successful strategy to obtain gene targeting in rice. To cover such approaches, we produced *ScRAD54* and *PpRAD51* transgenic barley plants and analyzed gene targeting with them. In addition, we adapted the rice P-N selection system to barley and tested it in barley.

## Materials and Methods

### Isolation of the Barley Golden Promise *ALS* Gene

**FIGURE 1:**
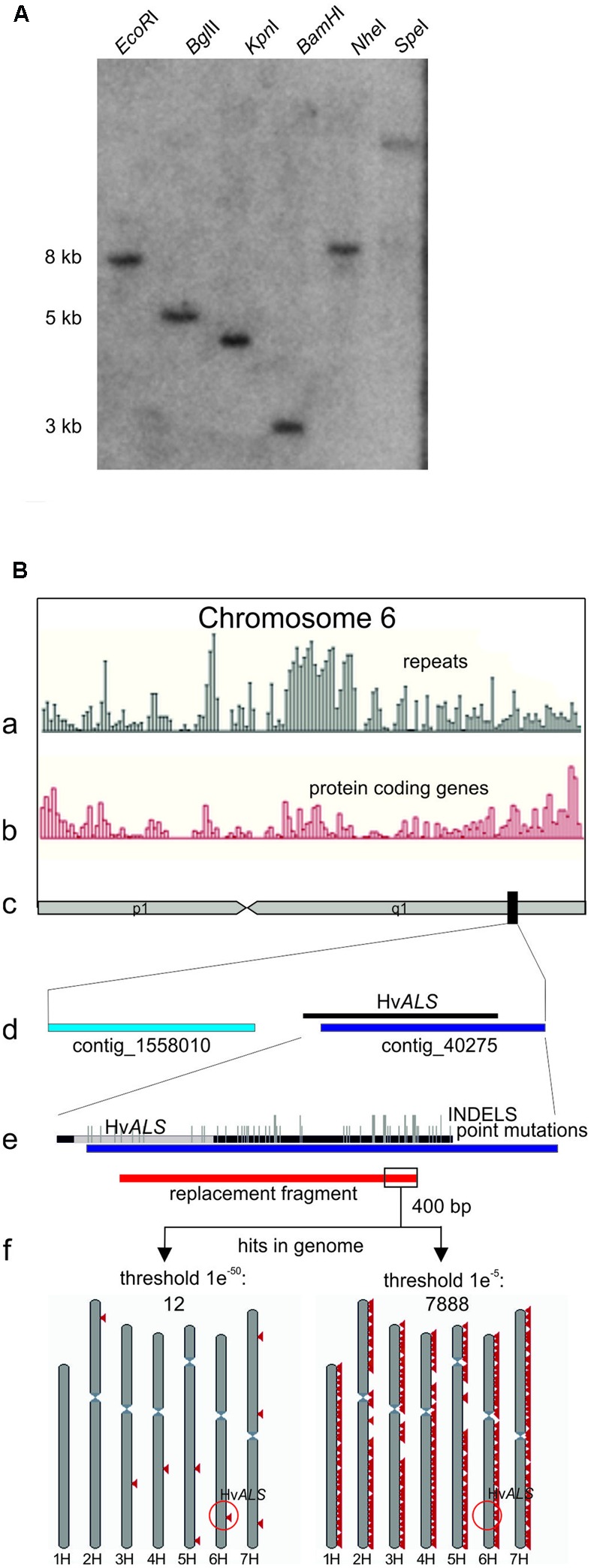
**The barley Golden Promise *ALS* gene. (A)** Southern blot showing that *ALS* is a single copy gene in barley. Genomic Golden Promise DNA was digested with the enzymes indicated and the blot probed with a Hv*ALS* fragment covering the carboxy-terminal region. A single fragment is detected with all enzymes confirming that *ALS* is a unique gene. **(B**) Bioinformatic analysis of the *ALS* gene locus. **(c)** Chromosome 6H (as depicted in the whole genome assembly (Gramene Hordeum vulgare assembly ASM32608v1^1^) is shown schematically in the center of the sketch. The position of *ALS* is marked by a black rectangle. The distribution of repeats **(a)** and protein coding regions **(b)** on chromosome 6H (Gramene database) is shown above. An enlargement of the region marked by the black rectangle containing *ALS* is shown below **(d)**. The enlargement shows the two anchored contigs 1558010 and 40275 located in this area together with a schematic alignment to the 5 kb of *ALS* sequence from Golden Promise. The same alignment at higher magnification is shown below **(e)** and displays the sequence divergence between the cultivars Golden Promise and Morex and exact position of the *ALS* coding region in gray. Point mutations are indicated by short vertical bars; INDELS by long vertical bars. **(f)** Design and results of the BLAST analysis. The position of the gene replacement fragment is shown in red below the alignment in **(e)**. The 400 bp 3′ terminal nucleotides used as query are boxed. The BLASTN results with two different threshold settings are shown schematically below. Red triangles mark hits on the seven barley chromosomes which are shown in gray. Triangles may represent more than one hit. The e values refer to the threshold settings in the BLASTN analysis^1^. The position of *ALS* on chromosome 6H is highlighted (red circle).

A BLAST search with the rice ALS protein sequence (GI: 189031230) as query identified 13 entries (HB27A17r, HF08O07r, HF22F02r, HH04G02u, HO28K09S, HO28K09w, HQ01F18w, HS06N21r, HS17M10u, HS18N04r, HS18N04u, HT06N21r, and RUS50B01w) in the barley CR-EST database (IPK Gatersleben) and five in the EMBL-EBI ENA database (AF059600, HQ661102, HQ661103, AK361384, and AK368472). The sequences from both databases assembled into one contig. A PCR product obtained with primers ALSF (CAT GTC TCC ATT TGT GCA G) and ALSR (CTG CCA TCA CCC TCC ATG) and EST clone HQ01F18w as template was used to probe a Southern blot prepared from Golden Promise genomic DNA digested with the enzymes indicated in **Figure 1A**. The 5 kb region in the *Bgl*II digest visible in **Figure 1A** was excised from an agarose gel run in parallel, and a sub-genomic library was prepared (Seidman, 2001) by ligation into *BamH*I digested pBSK- vector (Stratagene). pBSK-ALS, the plasmid carrying the 5 kb fragment with the Golden Promise *ALS* gene was identified by PCR screening in pools of transformants. The insert was sequenced in both strands by Sanger sequencing. The DNA sequence is available at the European Nucleotide Archive (ENA) under accession number LT601589.

### Molecular Methods

Genomic DNA was prepared as described (Dellaporta et al., 1983) or by the Qiagen Plant DNA easy kit (Qiagen, Hilden, Germany) as described by the manufacturer. PolyA^+^ RNA was extracted from leaf tissue using the Dynabeads mRNA DIRECT Kit (Invitrogen). Southern and Northern blotting was as described (Markmann-Mulisch et al., 2007). For Western blots, samples were prepared by crushing leaves and boiling the extract directly in sample buffer. The blots were prepared as described (Sambrook et al., 1989). Rabbit anti PpRAD51B antibody was obtained by commercial immunization (BioGenes, Gesellschaft für Biopolymere, Berlin) with purified protein overexpressed in *E. coli* (Ayora et al., 2002). The PCR analysis of gene targeting was done with Taq Polymerase (Ex Taq, Takara/ClonTech Europe) as described by the manufacturer using 35 cycles, denaturing temperature 98°C, annealing temperature 64°C, extension temperature 72°C. Primers were: PCR1, m567 (CCA TCA CCA AGC ACA ACT ACC TGG), m564 (GGT CAG CCG ACA ACT CTG AGG; PCR2, m567, m566 (GAG TGT CGT GCT CCA CCA TGT TG); PCR3, p35Sfwd (ACG CAC AAT CCC ACT ATC CTT C), m570 (CCG GAT CGG ACG ATT GCG TC).

### Vector Constructions

#### Construction of p35S-ALS^S629N^

A binary vector, pH001-ALS^S629N^ carrying a Pursuit resistance conferring *ALS* gene was constructed. To obtain this plasmid, a *Ssp*I/*Bam*HI fragment from pBSK-ALS containing the entire *ALS* coding region was fused to the maize *ubiquitin* (*ubi*) promoter (Christensen and Quail, 1996), inserted into the cloning vector pBSK- and then transferred into pH001-VS, a pS001 derivative (Reiss et al., 1996) in which the RK2 origin of replication was replaced with the one of pVS1 and the Sulfonamide resistance gene exchanged for the intron-split hygromycin resistance gene of pWBVec8 (Wang et al., 1998). The S629N mutation was introduced into the *ALS* gene by oligo-directed mutagenesis using PCR and oligos S629N PmlI FWD (GAG CAC GTG CTG CCT ATG ATC CCA AAC GGT GCT TTC AAG GAC) and S629NSbfIREV (GGC ATG CAC ATA CAA ATG GAC) and replacement of the wild type with the mutant sequences on the *Pml*I/*Sbf*I fragment. Since pH001-ALS^S629N^ carried a Carbenicillin resistance gene for selection in *Agrobacteria* and could therefore not be used with AGL1 (Wang and Waterhouse, 2000), p35S-ALS^S629N^, the plasmid finally used for transformation was constructed by exchanging the original vector for pWBVec8 and replacing the *ubi* promoter with the CaMV 35S promoter (Odell et al., 1985). To obtain this plasmid, the *Pst*I/*Bam*HI fragment from pH001-ALS^S629N^ carrying the *ALS*^S629N^ gene, an *Eco*RI/*Pst*I fragment carrying the 35S promoter and a *Bam*HI/*Hind*III fragment containing the 35S polyadenylation signal from pDH51 (Pietrzak et al., 1986) were inserted between the *Not*I and *Hind*III sites of pWBVec8.

#### Construction of *PpRAD51B* and *ScRAD54* Overexpression Vectors

pPEX002-VS is a pH001-VS derivative carrying the *phosphinotricin acetyltransferase* (*pat*) gene from pRT77 (Toepfer et al., 1993) instead of the *hpt* gene and an expression cassette consisting of the 35S promoter and polyadenylation signal from pDH51 (Pietrzak et al., 1986) at the right border. Vector pPEX-RAD51 was obtained by replacement of the 35S with the *ubi* promoter and insertion of the *PpRAD51B* gene from a cDNA clone (accession Nr. AJ316538, Markmann-Mulisch et al., 2002) into the cassette. Plasmid pPEX-RAD54 was obtained the same way except that the yeast RAD54 gene from pHS-35SRAD54 (Shaked et al., 2005) was inserted.

#### Construction of Gene Targeting and P-N Selection Vectors

All gene targeting plasmids go back to one common progenitor, pGT-0ALS^mS629N^. This plasmid harbours the fully assembled gene replacement fragment as a *Sal*I/*Xma*I fragment in a binary vector (pMin) with minimalised T-DNA border sequences, a vector backbone consisting of plasmid pVS1, a ColE1 origin, an Ampicillin/Carbenicillin resistance gene and a small polylinker with *Hind*III, *Sal*I, *Xma*I, and *BamH*I sites between the right and left T-DNA border sequences. The plasmid was constructed in detail as follows: First, a targeting vector version carrying a S629I mutation was constructed (pGT-0ALS^mS629I^). The 5′ truncated *ALS* gene was assembled from two PCR fragments, both amplified from pBSK-ALS as template. The upstream part was amplified with the primer ALS-SalI-FWD (GTG GTC GAC TCG CGT CCT CTG GCC GCC CGG GG) incorporating an artificial *Sal*I restriction site and ALS-PmlI-REV (GCA GCA CGT GCT CCT GAT GCG GGA CAA TGA TAT CCA GCA GGT AGG GCC CTG GGG TCT CAA GCA TC) which contained the natural *Pml*I site and incorporated the G to C diagnostic mutation which creates an artificial *Apa*I restriction site. The downstream part was obtained with primers ALS-PmlI-FWD-S629I (GAG CAC GTG CTG CCT ATG ATC CCA ATC GGT GGT GCT TTC AAG GAC) which contained the same natural *Pml*I site and introduced the resistance mutation (S629I) in the *ALS* coding sequence and ALS-BamHI-ApaI-REV (CGC GGG CCC CAG GAT CCC AGC ACA CAC GAA TG) which contained the natural *BamH*I site in the sequences downstream of the *ALS* stop codon and introduced an artificial *Apa*I site. Both fragments were cloned into pGEM-T easy (Promega). The resulting plasmids were sequenced by Sanger sequencing to confirm the identity of the cloned sequences.

The pGEM-T based vector pBIM was generated containing a polylinker with *Apa*I, *BspH*I, *Spe*I, *Sal*I, *Nhe*I, and *Bgl*II sites. The intron-split hygromycin resistance gene was excised from pWBVec8 as *Xba*I-*Nhe*I fragment and inserted between the *Spe*I and *Nhe*I sites of pBIM. To obtain the 5′ portion of the targeting fragment, the two PCR fragments were excised from the intermediate by *Sal*I and *Pml*I or *Pml*I and *Apa*I, respectively, and inserted together with the intron-split *hpt* gene obtained as *Apa*I-*Bgl*II fragment from the intermediate into the *Sal*I and *Bgl*II sites of pBIM. Finally, this preassembled 5′ portion was excised as a *Sal*I-*Bgl*II fragment and inserted together with a *BamH*I-*Age*I fragment obtained from pBSK-ALS containing the 3′ portion into *Sal*I and *Xma*I digested pMin.

The S629N version was prepared by fragment substitution in the vector carrying the 5′ portion. A fragment carrying the S629N mutation was produced by PCR with primers ALS-S629N-PmlI-FWD (GAG CAC GTG CTG CCT ATG ATC CCA AAC GGT GGT GCT TTC AAG GAC) and ALS-MluI-REV (TGA TAT TCT TGG AGT AGA CGA G) and pBSK-ALS as template. The PCR product was cloned into pGEM-T easy and sequenced. Then the authentic *Pml*I-*Mlu*I fragment was exchanged with the one carrying the S629N mutation and the final vector assembled as the S629I version before. All cloning steps were verified by DNA sequencing.

To obtain pGT-1ALS^mS629N^, the gene targeting fragment of pGT-0ALS^mS629N^ was excised with *Xho*I (0.4 kb 3′ from the end of the *ALS* sequence) and *Cfr9*I, ligated to *Cla*I/*Cfr9*I (CGA TCC AAG ATC TTG GC/CCG GGC CAA GAT CTT GGA T) and *Eco*RI/*Xho*I (AAT TCC CAA CTA GTT GGC/TCG AGC CAA CTA GTT GGG) adapters and inserted between the *Eco*RI and *Cla*I sites of pWBVec8. To obtain pGT-2ALS^mS629N^, the *Ampicillin*/*Carbenicillin resistance* gene in the original vector was exchanged for a *Streptomycin resistance* gene and the *Sal*I/*Xho*I targeting fragment of pGT-0ALS^mS629N^ inserted into the *Sal*I site present in the polylinker of this plasmid. To obtain pINA-ALS^mS629N^, the *SalI* site in pGT-0ALS^mS629N^ was converted to *Spe*I by sub-cloning in pBSK- and the resulting *Spe*I/*Xho*I targeting fragment inserted between the *Spe*I and *Hind*III sites of pINA134, after conversion of the *Hind*III and *Xho*I sites to blunt ends.

### Barley Culture and Transformation

For transformation, binary vectors pPEX-RAD51 and PEX-RAD54 were transformed into *Agrobacterium* strain AGL0, all others into AGL1 (Wang and Waterhouse, 2000). The corresponding strains were: p35S-ALS^S629N^, A29; pGT-1ALS^mS629N^, A27; pGT-2ALS^mS629N^, A33; pINA-ALS^mS629N^, A28; pWBVec10, Vec10. The barley *cultivar* Golden Promise was the genotype transformed throughout. Plant growth and transformation was essentially as described (Jacobsen et al., 2006; Watanabe et al., 2016). Selection conditions were: hygromycin: 50 mg/l; PPT: 8 mg/l; and Pursuit (application ready formulated herbicide solution, BASF): 400 nM. All tissue culture media were as described (Jacobsen et al., 2006) except that casein hydrolysate (Hydrolysate N-Z-Amine-A) was omitted. The leaf painting assay (Wu et al., 2003) was used for segregation analyses of greenhouse grown plants as described (Jones and Sparks, 2008).

For the analysis of transient expression assays, immature embryos were co-cultivated with Vec10 for 2 days and stained for β-glucuronidase (GUS) expression as described (Suter-Crazzolara et al., 1995).

For the radiation resistance assays, homozygous GP-RAD51-1 and Golden Promise seeds were harvested and irradiated dry at the IAEA Terrestrial Environment Laboratory in Seibersdorf, Austria. Seeds were sown on soil, grown in an environmental chamber and evaluated after 4 weeks.

## Results

### The Barley *Acetolactate Synthase (ALS)* Gene

The barley *ALS* gene was identified in the CR-EST (IPK Gatersleben) and EMBL-EBI ENA databases by protein sequence homology searches with the rice gene as query. The corresponding DNA sequences were assembled into one contig and this information used to obtain a partial *ALS* sequence from EST clone HQ01F18w by PCR. A Southern blot was prepared from Golden Promise DNA and probed with this fragment. The results showed that *ALS* is a single copy gene in barley (**Figure 1A**). The genomic portion located on a *Bgl*II fragment was isolated, cloned and sequenced. The 5403 bp *ALS*-*Bgl*II fragment contained the complete *ALS* coding region including 277 bp upstream and 3185 bp downstream sequences. A comparison of this sequence to the published barley genome (International Barley Genome Sequencing Consortium et al., 2012) is shown in **Figure 1Be**. The sequence comparison revealed a remarkable number of differences between Golden Promise and the reference genome Morex, both in the coding (point mutations) and the non-coding regions (insertions/deletions) in this part of the genome.

A sequence comparison to the whole genome assembly available in the Gramene database^1^ (Hordeum vulgare assembly ASM32608v1) revealed the location of *ALS* on the long arm of chromosome 6H (**Figure 1Bc**). A closer inspection showed that the *ALS* gene is located on contig_40275, except its 5′ region (**Figure 1Bd**). The 5′ part was also not found in the anchored neighboring contig_1558010 suggesting the existence of a large gap between the two contigs which is not bridged by our sequence. The Gramene database also provides data on the distribution of protein coding and repetitive sequences across all chromosomes (**Figures 1Ba,b**). A closer inspection of chromosome 6H in this respect showed an enrichment of protein coding genes in the region around the *ALS* gene, as expected from its position (International Barley Genome Sequencing Consortium et al., 2012; Higgins et al., 2014). However, this analysis also revealed the presence of repeats in this area which co-localize with genes. The presence of repetitive DNA directly next to the *ALS* gene was confirmed by an additional analysis which used the 3′ terminal 400 bp of the gene replacement fragment (described below) as a query in a BLASTN search against the entire barley genome. A high (1*e*^-50^) threshold value (as defined in the Gramene database) setting identified sequence elements of 100 to 150 bp in length with almost perfect homology at eight other positions and on different chromosomes. A low threshold setting (1*e*^-5^) showed that shorter and less conserved sequences exist thousands of times in the genome (**Figure 1Bf**). These data indicate that the terminal 400 bp of the gene targeting fragment are sequences with interspersed repetitive DNA.

### *ALS* As a New Selectable Marker for Barley Transformation

**FIGURE 2:**
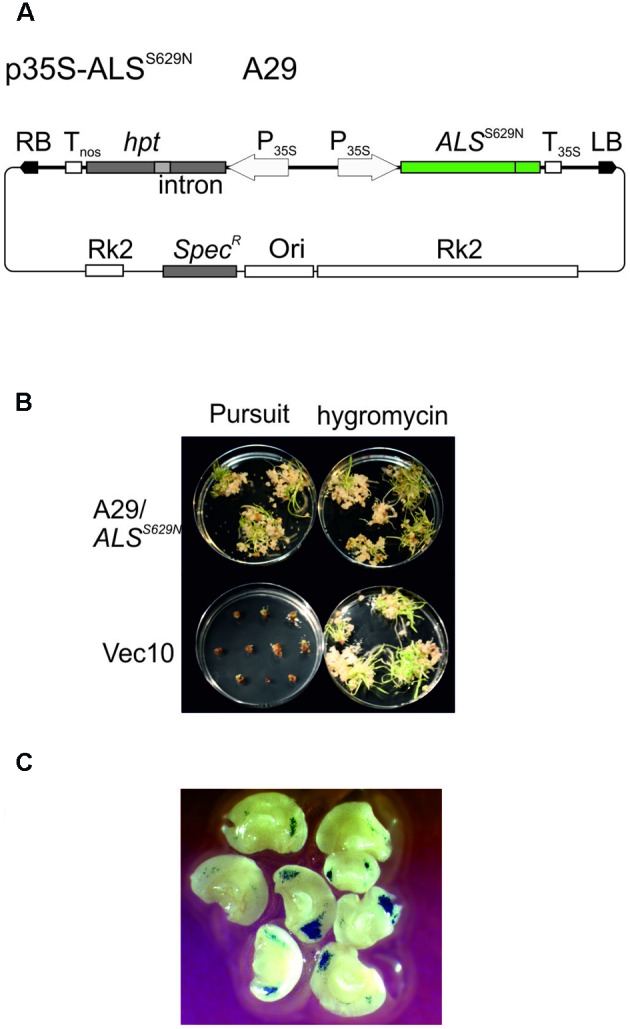
**Barley transformation and Pursuit selection. (A)** Binary vector p35S-ALS^S629N^ (strain A29) carrying an intact, Pursuit-resistance conferring *ALS* and the intron-split *hygromycin phosphotransferase* (*hpt*) gene. **(B)** Comparison of Pursuit and hygromycin selection. The pictures show the material obtained with co-cultivation of immature embryos with p35S-ALS^S629N^ (strain A29, upper panel) and pWBVec10 (strain Vec10, lower panel) at the end of the shoot induction period obtained with Pursuit (left column) and hygromycin (right column) selection on modified media. The picture shows that p35S-ALS^S629N^ transformed material grows equally well on Pursuit and hygromycin while Vec10 transformed material dies at the callus stage on Pursuit, but forms shoots on hygromycin. **(C)** Transient GUS expression obtained with Vec10 co-cultivated immature embryos after 2 days. The picture shows a large number of blue-stained spots indicating GUS gene expression on immature embryos. Abbreviations: RB, T-DNA right border; LB, T-DNA left border; *hpt* intron-split *hygromycin phosphotransferase* gene; Tnos, *nopaline synthase* gene terminator; P_35S_, CaMV 35S promoter; *ALS^S629N^* barley *acetolactate synthase* gene carrying the S629N mutation; T_35S_, CaMV 35S terminator; RK2, RK2 origin of replication and transfer region; Ori, ColE1 origin of replication; *Spec^R^*, *Spectinomycin resistance* gene.

Wild type ALS is inhibited by several different herbicides, one of which is the imidazolinone herbicide Imazethapyr, commercially available as Pursuit (BASF). A gene targeting assay system was developed for *A. thaliana* which is based on an *ALS* gene with a single point mutation at position 653 causing a serine to asparagine substitution and selection for Pursuit resistance (Badur and Reiss, 2004). The same mutation exists in imidazolinone herbicide resistant *ALS* genes of rice (Endo et al., 2006) and barley (Lee et al., 2011). This mutation was introduced into the Golden Promise Hv*ALS* gene by site directed mutagenesis. The homologous position in the barley protein sequence is S629 and conversion of the AGC codon at this position into AAC resulted in the desired S629N mutation. To express the Pursuit resistant *ALS* gene in barley, Hv*ALS*^S629N^ was placed between the CaMV 35S promoter (Odell et al., 1985) and polyadenylation signal (Sanfacon et al., 1991) and inserted into the barley transformation vector pMBVec8 (Wang et al., 1998). The resulting binary vector, p35S-ALS^S629N^ (**Figure 2A**) also harbors the intron-split *hygromycin phosphotransferase* (*hpt*) gene of pMBVec8 for selection on hygromycin. Vector p35S-ALS^S629N^ was transformed into *Agrobacterium* strain AGL1 to yield strain A29. For transformation control purposes, pMBVec10 (Wang and Waterhouse, 2000) with the same *hpt* gene and vector backbone as p35S-ALS^S629N^ was transformed into AGL1 to yield strain Vec10.

Barley Golden Promise transformation comprises co-cultivation of immature embryos with *Agrobacteria* (Tingay et al., 1997) followed by callus, shoot, and root induction on different media and under different conditions. The callus induction phase is the most critical period for selection. Embryos regenerated on the published callus induction media (Jacobsen et al., 2006) were barely sensitive to Pursuit at concentrations of 300, 600, and 1500 nM. Barley tissue culture media contain high concentrations of amino acids in the form of casein hydrolysate which could compensate for ALS inhibition by Pursuit. Therefore casein hydrolysate was omitted from the media and the inhibitory effect of Pursuit tested at concentrations of 50, 150, 300, 400, 600, 900, 1500, and 3000 nM. Callus and shoot growth were still observed at concentrations up to 300 nM, but almost completely inhibited at 400 nM Pursuit. To exclude that the omission of casein hydrolysate had any effect on callus induction and transformation efficiencies, immature embryos were transformed with Vec10 and selected on hygromycin using media with and without casein hydrolysate. The direct comparison of callus induction and transformation efficiencies showed that omission of casein hydrolysate had no effect. To characterize Pursuit selection further, immature embryos were co-cultivated with A29 and Vec10 and one half of them selected on the modified media containing either 400 nM Pursuit or hygromycin. The comparison of growth on Pursuit and hygromycin showed that both procedures perform equally well. With A29, callus and shoot development was comparable on Pursuit and hygromycin while Vec10 transformed calli were severely inhibited in growth on Pursuit and gradually showed brownish discoloration (**Figure 2B**). The transformation efficiencies obtained with A29 on hygromycin and Pursuit were 44 and 34%, respectively. Those with Vec10 were 39 and 0%, respectively, on hygromycin and Pursuit (**Table 1**). The data show that the transformation efficiencies obtained with A29 on Pursuit and hygromycin are the same within the variability of the experiments. The same is true for hygromycin selection and strains Vec10 and A29 which shows that both vectors performed equally well.

**Table 1:** Transformation efficiencies obtained with Pursuit and hygromycin selection.

	pMBVec10		p35S-ALS^S629N^	
Transformation experiment	hygromycin	Pursuit	hygromycin	Pursuit
1	40%	0%	45%	15%
2	65%	0%	50%	55%
3	25%	0%	35%	35%
4	25%	0%	45%	30%
Average	39±16%	0%	44±5%	34±14%

*Immature embryos were co-cultivated with the strains indicated and equal aliquots selected in the presence of hygromycin or Pursuit. Embryos giving rise to shoots that formed roots upon selection were counted as independent transformants. Transformation efficiency was calculated by dividing the number of independent transformants by the number of embryos transformed.*

The transformation efficiencies in these experiments were calculated as follows: A single, co-cultivated immature embryo usually yields a large, but variable number of shoots at the end of a transformation experiment. These can be of clonal origin or originate from different independent transformation events. To avoid ambiguities in the data, we counted only one shoot per embryo and defined an independent transformant as an embryo that produced at least one shoot forming a root in the presence of selective agent. Consequently, transformation efficiency is the number of immature embryos that formed shoots divided by total number of co-cultivated embryos.

The disadvantage of this definition is that it underestimates the true number of transformation events obtained in an experiment. To address this problem, we analyzed transient GUS expression in immature embryos co-cultivated with Vec10. These experiments (**Figure 2C**) confirm earlier observations (McCormac et al., 1998; Murray et al., 2004) showing that a large number of different cells is infected and at least transiently transformed by *Agrobacterium* in this procedure. Although clearly not all cells showing transient GUS expression give rise to stable transformants later, this assay is a reasonable proxy for transformation, and the data obtained with it suggest that a single immature embryo produces multiple independent transformation events.

A severe problem in barley transformation is overgrowth by *Agrobacteria*. Hygromycin selection was optimized to prevent overgrowth by *Agrobacteria*. The introduction of an intron-split *hpt* gene preventing its expression and the sensitivity of *Agrobacteria* to hygromycin largely solved the problem (Wang et al., 1998). However, there is no such system in Pursuit selection. Consequently, overgrowth was occasionally a larger problem with Pursuit selection that has caused loss of regenerating embryos. The problem was negligible with AGL1 provided the plant material was of good quality. However, the problem was quite severe with a different vector, pH001-ALS^S629N^ and strain AGL0. Many immature embryos were lost on Pursuit by overgrowth in this combination, suggesting that AGL1 is important for the performance and efficiency of Pursuit selection in barley.

### Establishment of *PpRAD51* and *ScRAD54* Overexpressing Barley Lines

**FIGURE 3:**
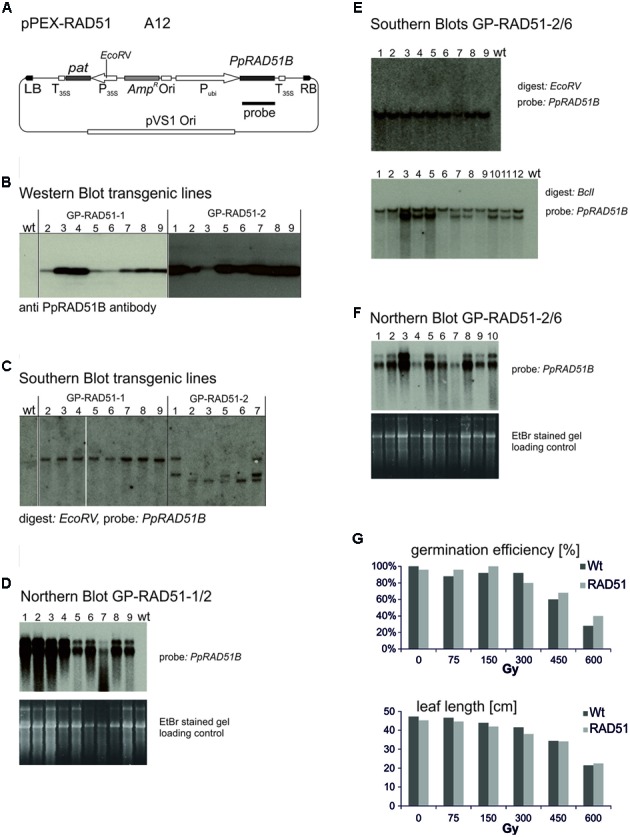
**Establishment and characterisation of PpRAD51B overexpressing lines. (A)** The vector pPEX-RAD51 (strain A12) used for transformation to obtain the overexpressing line. **(B)** Western blots. Protein extracts obtained with GP-RAD51-1 and GP-RAD51-2 progeny plants were separated by SDS acrylamide gel electrophoresis and the blot developed with anti PpRAD51B antibody. Lane numbers correspond to lines. **(C)** Southern blots. Genomic DNA prepared from the same plants was digested with *EcoR*V, separated by agarose gel electrophoresis and the blot probed with the radioactively labelled *PpRAD51B* fragment indicated in **(A)**. **(D)** Northern blot. Poly A^+^ RNA prepared from GP-RAD51-1/2 progeny plants was separated by formaldehyde agarose gel electrophoresis, the gel stained with EtBr and photographed (lower panel). The gel was blotted and the membrane probed with the radioactively labelled *PpRAD51B* fragment indicated in **(A)** (upper panel). **(E)** Southern Blot GP-RAD51-2/6. DNA of plants obtained from GP-RAD51-2/6 seeds DNA was digested with *EcoR*V or *Bcl*I and the blots probed with the radioactively labelled *PpRAD51B* fragment indicated in **(A)**. Lane numbers correspond to line numbers. **(F)** Northern Blot. PolyA^+^ RNA was prepared from the same population as in **(E)**, separated by formaldehyde agarose gel electrophoresis, the gel stained with EtBr and photographed (lower panel). The gel was blotted and the membrane probed with the radioactively labelled *PpRAD51B* fragment indicated in **(A)** (upper panel). **(G)** Radiation resistance assays. Homozygous GP-RAD51-1 and wild type Golden Promise seeds plants were γ-irradiated, grown on soil and germination rates or leaf length determined. The data show the average obtained from 25 seeds. The results show that RAD51 expressing and wild type plants germinated and grew equally well. Abbreviations: RB, T-DNA right border; LB, T-DNA left border; P_35S_, CaMV 35S promoter; T_35S_, CaMV 35S terminator; Ori, ColE1 origin of replication; *pat*, *phosphinotricin acetyltransferase* gene; *Amp^R^*, *Ampicillin*/*Carbenicillin* resistance gene; P_ubi_, maize *ubiquitin* promoter; pVS1 Ori, origin or replication from pVS1; probe, sequence used to probe the Southern and Northern blots.

The vector constructed to express the *P. patens PpRAD51B* gene (Markmann-Mulisch et al., 2002), pPEX-RAD51 (**Figure 3A**) is described in Section “Materials and Methods.” Strain A12 was obtained by transformation into Agrobacterium AGL0. To produce the *PpRAD51B* overexpression lines, 60 immature embryos were co-cultivated with A12 and transformants selected on phosphinotricin (PPT) containing media. These experiments yielded 22 PPT resistant plants originating on eight different immature embryos. To identify the individuals with high PpRAD51B protein expression levels, primary transformants (T0) were analyzed by Western blotting and a PpRAD51B-specific antibody raised against purified protein overexpressed in *E. coli* (Ayora et al., 2002). This screen identified two transformants with high PpRAD51B expression levels, GP-RAD51-1 and GP-RAD51-2. These plants were self-pollinated and the progeny (T1) analyzed in more detail. To determine the number of independent transgene inserts in the genome, inheritance of PPT resistance in the segregating progeny was analyzed with the leaf painting assay (Wu et al., 2003). Sixteen out of 23 GP-RAD51-1 siblings were PPT resistant closely matching the expected ratio of 3:1 for Mendelian inheritance of a single gene. All 23 GP-RAD51-2 siblings were PPT resistant indicating the presence of multiple, independently segregating inserts. This generation was further analyzed by Southern blotting (**Figure 3C**). Genomic DNA was prepared, digested with *EcoR*V, an enzyme producing a single cut within the inserted transgene, blotted and the membrane probed with a *PpRAD51B* gene fragment. The results revealed the presence of a single transgene insert in GP-RAD51-1 and at least three independently segregating inserts in GP-RAD51-2. Western blotting of the same plants showed that all siblings with a transgene expressed PpRAD51B protein (**Figure 3B**). To identify homozygous GP-RAD51-1 individuals, PPT resistant T1 plants were self-pollinated and segregation of PPT resistance determined in the progeny. This analysis identified one homozygous line (line 2) with 100% PPT resistant progeny (T2). To obtain sufficient seeds for the gene targeting experiments, T2 plants were propagated by self-pollination and PpRAD51B expression confirmed by Northern blotting (**Figure 3D**) in the plants grown from them. For the Northern blot, Poly A^+^ RNA was prepared, separated by formaldehyde agarose gel electrophoresis, blotted and the membrane hybridized with the *PpRAD51B* gene probe. All plants expressed *PpRAD51B* confirming stable inheritance of an active gene in this generation. However, for unknown reasons mRNA as well as PpRAD51B protein expression seems to be variable.

For GP-RAD51-2, line 6 (T1) was chosen as a plant with an apparent single copy transgene integration. The plant was self-pollinated and siblings analyzed in more detail. Leaf painting showed that PPT resistance segregated in this population, but the transgene was present in all individuals, as shown by Southern blotting (**Figure 3E**), whether resistant or sensitive. To resolve this issue, a Southern blot with an enzyme (*Bcl*I) not cutting within the transgene was prepared. This blot showed the presence of two different insertions in line 6 (**Figure 3E**), one of which co-segregated with PPT resistance while the other one was present in all siblings. The corresponding Northern blot showed that all of them express the *PpRAD51B* gene (**Figure 3F**). These results indicate that GP-RAD51-2 line 6 is homozygous for a *PpRAD51B* expressing transgene and hemizygous for an additional transgene with a functional *pat* gene.

RAD51 overexpression in mammalian cells improved gene targeting and resistance to DNA damaging agents in some cases (Vispe et al., 1998; Yanez and Porter, 1999; Lundin et al., 2003) but not in others (Lambert and Lopez, 2000). To see whether RAD51 overexpression has an effect on radiation resistance in barley, homozygous GP-RAD51-1 seeds were gamma irradiated, and seed germination rates and leaf length at the seedling stage scored as parameters to judge resistance (**Figure 3G**). There was no significant difference between RAD51 overexpressing plants and wild type. Seeds germinated well up to a dose of 300 Gy and germination rates gradually dropped afterward. Leaf growth and development was affected by doses above 75 Gy with leaves becoming gradually shorter and growth stunted at higher doses. The data show that dry barley seeds are highly resistant to γ-rays and RAD51 expression does not seem to have an effect on resistance under these conditions. This result is different from that obtained with *ScRAD54* in similar resistance assays (Shaked et al., 2005), but consistent with the results obtained with the *Atrad51* mutant in *A. thaliana* which suggest that RAD51 is not involved in the repair of the type of DNA damage that γ-rays cause (Markmann-Mulisch et al., 2007).

**FIGURE 4:**
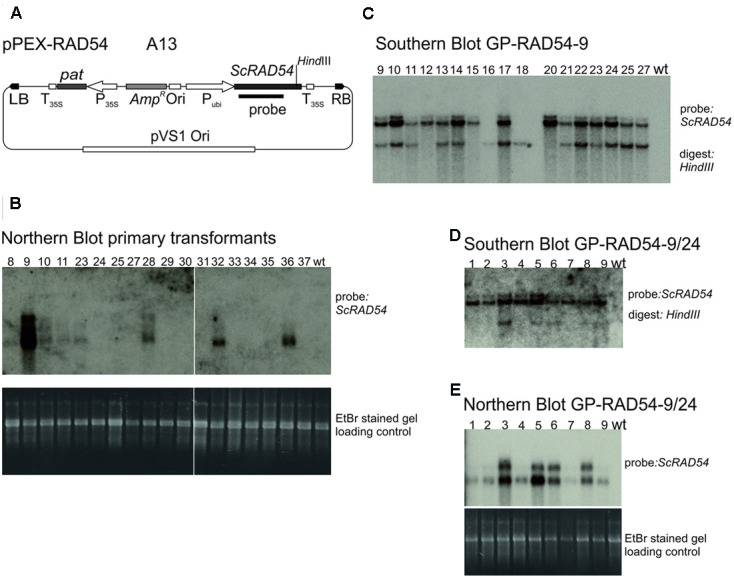
**Establishment and characterisation of ScRAD54 overexpressing lines. (A)** The vector pPEX-RAD54 (strain A13) used for transformation to obtain the overexpressing line. **(B)** Northern blots. Poly A^+^ RNA was prepared from primary transformants, separated by formaldehyde agarose gel electrophoresis, the gel stained with EtBr and photographed (lower panel). The gel was blotted and the membrane probed with the radioactively labelled *ScRAD54* fragment indicated in **(A)** (upper panel). **(C)** Southern blot GP-RAD54-9. Genomic DNA was prepared from GP-RAD54-9 progeny plants, digested with *Hind*III and the blot probed with the radioactively labelled *ScRAD54* fragment indicated in **(A)**. **(D)** Southern blot GP-RAD54-9/24. Genomic DNA was prepared from GP-RAD54-9/24 progeny, digested with *Hind*III and the blot prepared as in **(C)**. **(E)** Northern Blot GP-RAD54-9/24. Poly A^+^ RNA was prepared from the same plants as in **(D)**, separated by formaldehyde agarose gel electrophoresis, the gel stained with EtBr and photographed (lower panel). The gel was blotted and the membrane probed with the radioactively labelled *ScRAD54* fragment indicated in **(A)** (upper panel). Abbreviations: RB, T-DNA right border; LB, T-DNA left border; P_35S_, CaMV 35S promoter; T_35S_, CaMV 35S terminator; Ori, ColE1 origin of replication; *pat*, *phosphinotricin acetyltransferase* gene; *Amp^R^*, *Ampicillin*/*Carbenicillin* resistance gene; P_ubi_, maize *ubiquitin* promoter; pVS1 Ori, origin or replication from pVS1; probe, sequence used to probe the Southern and Northern blots.

The vector constructed to express the budding yeast *ScRAD54* gene (Shaked et al., 2005), pPEX-RAD54 (**Figure 4A**) is described in Section “Materials and Methods.” Strain A13 was obtained by transformation into Agrobacterium AGL0. To obtain *ScRAD54* overexpressing plants, 60 immature embryos were co-cultivated with A13 and transformants selected for PPT resistance. This transformation yielded 21 shoots originating on seven different immature embryos, 18 of which were viable and set seeds. These transformants were screened for *ScRAD54* expression by Northern blotting (**Figure 4B**) and the *ScRAD54* gene as probe. GP-RAD54-9, the primary transformant (T0) with the highest *ScRAD54* expression was self-pollinated and analyzed in the next generation by Southern blotting. Genomic DNA from plants in a segregating population was digested with *Hind*III cutting within the *ScRAD54* gene and the blot hybridized with the *ScRAD54* probe (**Figure 4C**). This blot indicated the presence of at least two independently segregating insertions in GP-RAD54- 9. Selected T1 plants were self-fertilized and the progeny tested for PPT resistance. The progeny of one plant (line 24) with 100% PPT resistant siblings was analyzed further. Northern blotting (**Figure 4E**) showed that all individuals expressed ScRAD54. Southern blotting showed that all had one transgene insertion in common (upper band in **Figure 4D**), while some of them carried an additional insertion (lower band in **Figure 4D**). An additional band appears on top of the upper band and is visible in most lanes and in both Southern blots (**Figures 4C,D**). This band is likely due to partial digestion, but could be another insertion. These results indicate that GP-RAD54-9/24 is homozygous for a transgene insertion harboring expressed *pat* and *ScRAD54* genes, and hemizygous for another insertion with unknown composition.

To get an estimate of how many different transformation events can be generated with one co-cultivated immature embryo, the Southern blots produced to characterize the overexpression lines were evaluated quantitatively. In this set, eight immature embryos produced a total of 23 shoots. Eighteen of these shoots differed in their integration pattern and therefore were independent transformants. Consequently, one transformed immature embryo produced 2.25 different transformation events on average in this sample.

### Analysis of Gene Targeting Using the *ALS* Gene

**FIGURE 5:**
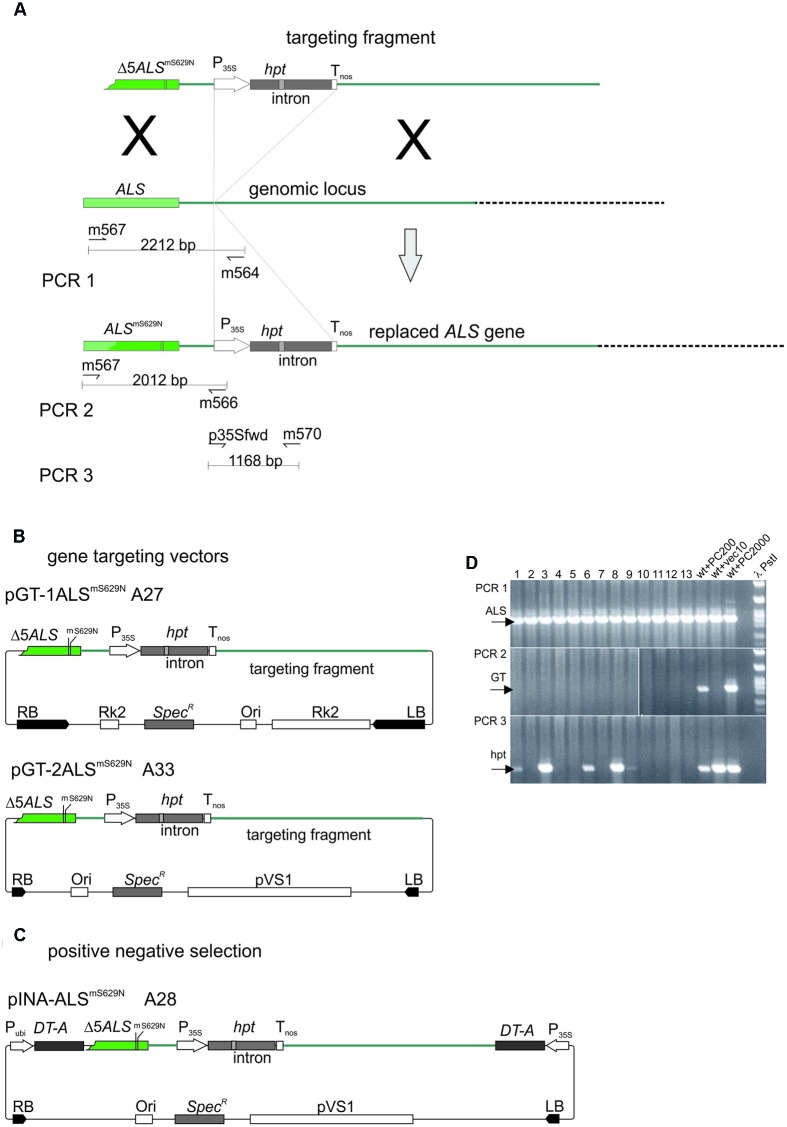
**The gene targeting experiments. (A)** Design of the gene targeting experiment and the PCR analysis. The genomic *ALS* gene target locus is shown in the center. The gene replacement fragment released from pGT-1ALS^mS629N^ and pGT-2ALS^mS629N^ is shown above. The recombination product obtained by precise gene replacement is shown below. Regions of homology are color coded and sites of potential crossovers indicated (X). The primers used in the PCR analysis are shown below the genomic *ALS* gene and the predicted gene replacement product. **(B)** The gene targeting and **(C)** P-N selection vectors used in this study. To highlight features in common, the gene targeting fragments as released with the T-DNAs are shown on top and the remaining parts below. Color codes are as in **(A)**. **(D)** Analysis of potential gene targeting events by PCR. Shoots obtained by Pursuit selection were collected in pools of 100, DNA prepared and analyzed by PCR. PCR1 amplifies a portion of the genomic *ALS* gene and the corresponding results are shown in the PCR1 panel. PCR2 amplifies a fragment overlapping the 5′ recombination junction and these results are shown in the PCR2 panel. PCR3 detects the *hpt* gene fragment and the corresponding results are in the PCR3 panel. Lanes 1–13, pools 1–13, wt+PC200, barley wild type DNA spiked with 200 molecules test construct; wt+vec10, barley wild type DNA spiked with 200 molecules pWBVec10; wt+PC2000, barley wild type DNA spiked with 2000 molecules test construct; bbb *Pst*I, *Pst*I digested phage lambda DNA. Abbreviations: RB, T-DNA right border; LB, T-DNA left border; Δ5*ALS*^mS629N^, 5′ truncated *ALS* gene with the S629N and diagnostic, silent (m) mutation; P35, CaMV 35S promoter; *hpt* intron-split *hygromycin phosphotransferase* gene; Tnos, *nopaline synthase* gene terminator; *ALS^mS629N^* barley *acetolactate synthase* gene carrying the S629N and diagnostic (m) mutations; RK2, RK2 origin of replication and transfer region; Ori, ColE1 origin of replication; *Spec^R^*, *Spectinomycin resistance* gene; P_ubi_, maize *ubiquitin* promoter; pVS1 Ori, origin or replication from pVS1; *DT-A*, *diphtheria toxin* gene.

The *ALS*-based gene targeting assay system established for barley is shown in **Figure 5A**. The design follows the one used to analyze gene targeting in *A. thaliana* before (Badur and Reiss, 2004; Prinzenberg, 2006). The system comprises a gene replacement fragment with a non-functional, 5′ terminally truncated *ALS* gene carrying the S629N mutation and a second, silent diagnostic mutation which allows independent identification of the introduced gene. Then an intron-split *hpt* gene under 35S promoter control follows which was inserted between the putative polyadenylation/termination signal of *ALS* and the following 2 kb of sequences present in the genome. This design provides 1.2 kb of sequence homology to the genome at the 5′ and 2 kb at the 3′ end of the replacement fragment. Two different gene targeting vectors (**Figure 5B**) were obtained by insertion of the same gene targeting fragment into two different vector backbones. In one of them, pGT-1ALS^mS629N^ the backbone was pMBVec8. For the other one, pGT-2ALS^mS629N^, a newly constructed vector with minimalised T-DNA border sequences was used to reduce heterology at the ends. Both plasmids were transformed into AGL1 resulting in strains A27 for pGT-1ALS^mS629N^ and A33 for pGT-2ALS^mS629N^.

To analyze gene targeting, immature wild type embryos were co-cultivated with A27 or A33 and transformants selected on Pursuit. To control the quality of each single transformation experiment, an additional aliquot of 20 immature embryos was co-cultivated in parallel with the same A27 or A33 *Agrobacterium* culture and selected on hygromycin. This reference transformation served to assure quality of individual transformation experiments and to determine the transformation efficiency obtained in each of them. Transformation efficiencies were determined as before except that rooting assays were omitted for hygromycin. However, they were continued for Pursuit selection. For wild type, 887 embryos were transformed with A27 or A33 and selected on Pursuit (**Table 2**). The reference transformations on hygromycin predicted that this number would have generated 465 independent transformants. A total of 130 calli formed shoots on Pursuit in these experiments, but none of them rooted in the presence of Pursuit later. Consequently, no Pursuit resistant transformant was among them and these experiments did not yield a single gene targeting event.

**Table 2:** Summary of gene targeting experiments in wild type and transgenic lines.

Exp.^(A)^	Vector	Number of immature embryos selected on Pursuit^(B)^	Transformation efficiency in hygromycin control^(C)^	Number of independent transformation events predicted for Pursuit selection^(D)^	GT^(E)^
**Wild type**

1	pGT-1ALS^mS629N^	100	60%	60	0
2	pGT-1ALS^mS629N^	100	65%	65	0
3	pGT-1ALS^mS629N^	100	50%	50	0
4	pGT-1ALS^mS629N^	158	60%	95	0
5	pGT-1ALS^mS629N^	93	63%	58	0
6	pGT-1ALS^mS629N^	247	40%	99	0
7	pGT-2ALS^mS629N^	89	43%	39	0

Total		887		465	0

**RAD51**

1	pGT-1ALS^mS629N^	100	70%	70	0
2	pGT-1ALS^mS629N^	100	70%	70	0
3	pGT-1ALS^mS629N^	200	55%	110	0
4	pGT-1ALS^mS629N^	160	85%	136	0
5	pGT-1ALS^mS629N^	180	90%	162	0
6	pGT-1ALS^mS629N^	218	55%	120	0
7	pGT-1ALS^mS629N^	213	47%	99	0
8	pGT-2ALS^mS629N^	100	80%	80	0
9	pGT-2ALS^mS629N^	160	70%	112	0
10	pGT-2ALS^mS629N^	120	35%	42	0
11	pGT-2ALS^mS629N^	50	90%	45	0
12	pGT-2ALS^mS629N^	100	65%	65	0
13	pGT-2ALS^mS629N^	160	75%	120	0

Total		1861		1231	0

**RAD54**

1	pGT-1ALS^mS629N^	82	56%	46	0
2	pGT-2ALS^mS629N^	54	50%	27	0

Total		136		73	0

*(A) Experiment number. (B) Immature embryos prepared from wild type, GP-RAD51-1/2 (RAD51) or GP-RAD54-9/24 (RAD54) plants. (C) In the reference transformation, 20 immature embryos were transformed in the same experiment with the same vector, selected on hygromycin and the transformation efficiency calculated by dividing the number of embryos forming at least one resistant shoot by the number of embryos transformed. (D) The number of independent transformants obtained on Pursuit was obtained by multiplication of the transformation efficiency in the hygromycin control (C) with the number of immature embryos selected on Pursuit (B). (E) The number of gene targeting (GT) events obtained.*

To analyze gene targeting in *PpRAD51B* and *ScRAD54* overexpressing lines, immature embryos obtained from plants grown from homozygous GP-RAD51-1/2 and GP-RAD54-9/24 seeds were co-cultivated with A27 and A33 and selected on Pursuit. The experiments included the same reference transformations as for wild type described above. In all transformation experiments together, 1861 RAD51 and 136 RAD54 transgenic embryos were transformed with A27 or A33 (**Table 2**). The reference transformation on hygromycin predicted that 1304 independent transformants were obtained on Pursuit altogether (**Table 2**). Although many calli produced shoots, none of them was a Pursuit resistant transformant.

The reference transformation also showed that the pGT-1ALS^mS629N^ (A27) and pGT-2ALS^mS629N^ (A33) targeting vectors performed equally well since both yield comparable transformation efficiencies on hygromycin, 62±23% and 72±14% (**Table 2**). Therefore, potential differences between the strains seem to be negligible and the data were pooled.

### Positive-Negative Selection

In P-N selection, the positive marker is used to select for the integration of the gene replacement fragment while the negative marker prevents random integration. A potent negative selection marker is the 35S-promoter-driven *diphtheria toxin-A* (*DT-A*) gene. Placed at the ends of the gene replacement fragment, these sequences are lost upon homology-mediated integration but maintained in random integration and cause cell death. To obtain a P-N selection system in barley, the *ALS^mS629N^* gene replacement fragment was inserted between the two *DT-A* genes flanking each T-DNA border region of pINA134 (Terada et al., 2002). The resulting vector, pINA-ALS^mS629N^ (**Figure 5C**) was transformed into AGL1 and the resulting strain (A28) used for co-cultivation of wild type embryos. In total, 1645 immature embryos were transformed and selected on hygromycin (**Table 3**). Transformation efficiencies were monitored by transforming an aliquot of the same batch with Vec10, selection on hygromycin and scoring callus growth. Compared to Vec10, A28 transformed calli developed considerably slower and poorer on hygromycin, as expected for negative selection. Very few calli produced poorly growing shoots, only one of which formed roots on hygromycin. Molecular analysis revealed later that this one was not targeted. The reference transformation with Vec10 suggests that 1270 independent transformants were generated in the P-N transformation experiments.

**Table 3:** Positive-negative selection.

Exp.^(A)^	Vector	Number of immature embryos in P-N selection on hygromycin^(B)^	Transformation efficiency in pMBVec10 control^(C)^	Number of independent transformation events predicted in P-N selection^(D)^	GT^(E)^
1	pINA-ALS^mS629N^	180	100%	180	0
2	pINA-ALS^mS629N^	284	58%	164	0
3	pINA-ALS^mS629N^	391	61%	239	0
4	pINA-ALS^mS629N^	280	100%	280	0
5	pINA-ALS^mS629N^	280	90%	252	0
6	pINA-ALS^mS629N^	230	68%	155	0

Total		1645		1270	0

*(A) Experiment number. (B) Immature embryos prepared from wild type were transformed with the P-N targeting vector pINA-ALS^mS629N^ and selected on hygromycin. (C) In the reference transformation, 20 immature embryos were transformed in the same experiment with pMBVec10 and selected on hygromycin. The transformation efficiency in the reference was calculated by dividing the number of embryos forming callus in the presence of hygromycin by the number of embryos transformed. (D) The number of independent transformants obtained by P-N selection was obtained by multiplication of the transformation efficiency obtained with pMBVec10 (C) with the number of immature embryos in the P-N selection (B). (E) The number of gene targeting (GT) events obtained.*

### Molecular Analysis of Gene Targeting Events

The *ALS*-based gene targeting assays rely on the formation of a functional, Pursuit resistance conferring gene. To exclude that selection problems have prevented rooting on Pursuit, all shoots obtained in the above assays were tested in pools of 100 by PCR. All gene targeting vectors used the same replacement fragment (**Figure 5**) and would create the same 5′ recombination junction upon HR with the *ALS* gene in the genome. This junction is detected specifically in PCR2 (**Figure 5A**). Diploid barley nuclei contain 5.79 pg of nuclear DNA. This number corresponds to 20.000 diploid genomes in the amount of DNA used in a standard PCR reaction (120 ng), or 200 genomes that one individual contributes to a pool of 100 transformants. To optimize the PCR reaction, serial dilutions of a control plasmid which contained a 5′ recombination junction generated *in vitro* by cloning were mixed with 120 ng genomic Golden Promise DNA and conditions optimized until 200 recombination junction molecules were reliably detected. Additional PCRs were used to detect the genomic *ALS* gene (PCR1) and the *hpt* gene in the gene replacement fragment (PCR3).

Shoots obtained by Pursuit or P-N selection were collected in pools of 100, DNA prepared and amplified by PCR2. The corresponding product was not present in any of the pools, but it was easily amplified from the control DNA (**Figure 5D**). The same was true for the single hygromycin resistant shoot obtained by P-N selection. Since PCR1 showed that all DNA preparations were PCR-amplifiable (**Figure 5D**) the results show that the 5′ recombination junction was not present in any of the transformed plants. In addition, many of the pools did not even contain transformants since the *hpt* gene detected with PCR3 was not present in all them. By contrast, the same fragment was present in all 33 individual A27 transformants selected on hygromycin.

## Discussion

The *ALS* gene including the flanking regions was isolated from barley cv. Golden Promise and compared to the barley reference genome sequence obtained from cv. Morex (International Barley Genome Sequencing Consortium et al., 2012). The sequences show that both genomes are largely identical in this region, but an astonishing number of point mutations in the coding sequence and additionally deletions and insertions in the flanking regions exist (**Figure 1Be**). These data suggest a considerable divergence of the Golden Promise and Morex genomes, as observed with other cultivars (International Barley Genome Sequencing Consortium et al., 2012) and implied by the existence of a large number of polymorphic markers (Rae et al., 2006). These differences suggest that the barley genome is still quite dynamic. In particular, the frequent occurrence of INDELS in non-coding regions point to the importance of DNA damage repair by erroneous pathways in barley, as suggested (Vu et al., 2014). In addition, the high degree of sequence divergence between cultivars could have impacted gene targeting efficiencies if isogenic *ALS* gene DNA had not been used.

The Pursuit resistance conferring *ALS* gene is a useful, new selectable marker for transformation of barley in tissue culture. The number of established selectable markers is currently restricted to *hpt* and *pat*. The optimized conditions for Pursuit selection, or structurally similar imidazolinone herbicides and the availability of the *ALS* gene add a valuable third alternative with an overall performance comparable to the unparalleled hygromycin selection.

In the gene targeting experiments, 887 wild type, 1861 *PpRAD51B*, and 136 *ScRAD54* transgenic immature embryos were transformed with the *ALS* gene-based replacement vectors and selected for Pursuit resistance. Additional 1645 wild type embryos were transformed with the P-N construct and selected on hygromycin. Together, these are 4529 embryos that were screened for gene targeting, either using the *ALS* gene and selection for Pursuit resistance or by P-N selection. None of these experiments yielded a single gene replacement event. Technical reasons potentially contributing to this result can be excluded. Each transformation experiment included transformation and selection (hygromycin and Pursuit) controls. The experimental design of the gene targeting assay system closely follows the one successfully used in *A. thaliana* before. This includes the choice of the resistance mutation, the selection conditions and the design of the gene replacement vector. The conditions used for Pursuit selection were carefully adapted to barley. To do this, the Pursuit concentration minimally necessary to inhibit callus growth was determined and a concentration slightly higher (400 nM) used for selection later. The same strategy was used in *A. thaliana* before and a comparable Pursuit concentration (300 nM) allowed detection of gene targeting there. An endogenous *ALS* gene carrying the same mutation confers resistance to imidazolinone herbicides in barley (Lee et al., 2011) testifying the functionality of the gene, even in heterozygous condition. This is also true for *A. thaliana* and here the gene confers resistance in callus tissue. Therefore, *ALS* is expressed in undifferentiated tissue and the resistant version confers resistance under these conditions. Both argue strongly against a malfunctioning *ALS* selection system as cause of failure. Last not least, the P-N experiments provide evidence independently from Pursuit selection that efficiencies are low, at least transformation of 1645 embryos did not yield a single event.

Transformation of 4529 immature embryos in all experiments together produced 3039 independent transformants. We counted an embryo as independent transformant when it produced a transformed shoot. However, many more shoots can be obtained from a single co-cultivated immature embryo. Transient expression experiments (McCormac et al., 1998; Murray et al., 2004) including our own (**Figure 3B**) suggested that these could be in the hundreds, but only few of them may be stable transformants. The number of independent transformants obtained from a single embryo is unknown, but our preliminary analysis suggested that 2.25 are generated with a single immature embryo on average. Taking this number, we analyzed 6838 potential transformants for gene targeting and did not obtain one single event.

How does barley compare to *A. thaliana* and rice? The assay system used in *A. thaliana* and barley is identical, except the transformation methods. The different transformation methods necessitate different reference transformations. In *A. thaliana*, root transformation is used and the number of single calli formed on the explants represents the real number of transformants obtained in an experiment. Using this system, 0.8 in 1000 transformants were Pursuit resistant and thus potential gene targeting events (Prinzenberg, 2006). Comparable efficiencies were found with a different target gene and a different gene targeting assay system (Hanin et al., 2001) suggesting that efficiencies in this order of magnitude are typical for *A. thaliana*. With our estimate of 6838 transformation events generated in the 4529 immature embryos, comparable gene targeting efficiencies in barley would have generated five events, but we expected many more since one immature embryo probably yields more than two transformation events. The differences between *A. thaliana* and barley become even more apparent with the PpRAD51B overexpression line. PpRAD51B overexpression improved the efficiency of gene targeting to three in 1000 transformants in *A. thaliana* (Prinzenberg, 2006). Transformation of 1861 *PpRAD51B* transgenic barley embryos produced an estimated 2770 transformants and eight of these should have been targeting events in this sample only. It remains possible that *PpRAD51B* expression has not stimulated gene targeting in barley. But even then the number of immature embryos transformed in these experiments adds to the total number of immature embryos analyzed for gene targeting.

An *ALS* gene-based assay based on comparable transformation methods (Toki et al., 2006) was also used in rice (Endo et al., 2007; Saika et al., 2015). In rice, 1500 embryos yielded 66 independent gene targeting events (4.4% of transformed embryos) (Endo et al., 2007) suggesting that high efficiencies of gene targeting are obtained at the *ALS* gene with the same transformation method. These high efficiencies are confirmed by P-N selection data. With a slightly different transformation method, P-N selection was a very efficient approach in rice and allowed the routine use of gene targeting for gene modification (Terada et al., 2002, 2007; Iida and Terada, 2004; Yamauchi et al., 2009; Shimatani et al., 2015). Transformation of 1645 immature embryos in barely produced a predicted number of 2857 transformants, but not a single gene targeting event was obtained. The same number of immature embryos would have produced 72 events in rice assuming an efficiency of 4.4% as described by Endo et al. (2007). These results provide independent evidence for the low efficiency. They also suggest that P-N selection is not an option to obtain gene targeting in barley within the limits of reasonably scaled experiments.

There are clear differences between barley, *A. thaliana* and rice. One of them is the transformation method. In rice, immature embryos or amplified embryogenic calli for P-N selection are transformed. Both, Pursuit and P-N selection positively select for gene targeting events among a potentially large number of transformants. Therefore, differences in the transformation methods might explain the differences between rice and barley. We cannot exclude that significantly more transformation events are generated in a co-cultivated immature embryos in rice as compared to barley. In this case, the same number of immature embryos simply generated more transformation events in rice than in barley. The gene targeting efficiencies would be overestimated in rice then, but are still sufficient to obtain gene targeting in P-N selection. However, transformation method-based differences cannot explain the differences to *A. thaliana*.

There are more explanations. Tobacco is the only other plant with a large genome for which data on gene targeting in the absence of DSB induction are available. The efficiencies in tobacco are also very low (Reiss, 2003), also with the *ALS* gene (Lee et al., 1990). Barley and tobacco have large, complex genomes with a high content of repetitive DNA. *A. thaliana* and rice genomes are small and gene-rich with minimal repetitive DNA. This suggests that gene targeting efficiency could be related to genome size or complexity, with plants with large genomes like barley and tobacco being inefficient and those with gene rich genomes efficient. Genome size alone could be the factor. Gene targeting could always occur at DSBs that naturally occur randomly during the live cycle in any genome. The number of DSBs could be the same in every genome, e.g., if they would occur in transcribed genes only. Then DSBs would occur less frequently at a given locus in plants with large genomes and consequently gene targeting efficiencies were lower.

Genome complexity, presence of repetitive DNA and differences in the mechanisms of HR provide alternative explanations. The barley genome contains ample repetitive DNA, preferentially located in the large centromeric or centromere proximal regions while the sub-terminal regions are relatively gene-rich areas. In addition, the position in the genome, whether in a gene or repeat-rich area could have an effect on gene targeting efficiencies, as it has on meiotic recombination (International Barley Genome Sequencing Consortium et al., 2012; Higgins et al., 2014). However, the position of *ALS* in a gene-rich subtelomeric region on the long arm of chromosome 6H (**Figure 1Bc**) makes this scenario unlikely. Nevertheless, repetitive DNA could still be the reason. Gene targeting in the absence of DSB induction in *P. patens* depends on a RAD51-dependent strand-invasion strand-exchange pathway leading to crossover recombination (Wendeler et al., 2015). Crossover recombination at ectopic positions, i.e., at homologous or highly related sequences present at different locations in the genome can cause genome rearrangements. To prevent genome rearrangements this sub-pathway of HR may generally be downregulated in plants with large, complex genomes to minimize ectopic HR. This risk is much lower in plants with small, gene-rich genomes and these could tolerate higher activities of crossover HR consequently resulting in higher gene targeting efficiencies. However, crossover recombination does occur in barley as shown by active sister chromatid exchange (SCE) (Vu et al., 2014). Yet, SCE may be a special case since crossover recombination is restricted to the sister chromatid here.

The low gene targeting efficiencies in the absence of DSB induction contrast the high efficiencies with DSB induction, in barley (Watanabe et al., 2016) and in tobacco (Townsend et al., 2009). This difference may be due to differences in the mechanisms used for gene targeting with and without a DSB. While crossover recombination likely mediates gene replacement in the absence of a DSB, synthesis-dependent strand annealing (SDSA), a DNA damage repair pathway avoiding genome rearrangements (Heyer et al., 2010), or possibly single-strand annealing (SSA), is used in plants when a DSB is present (Puchta et al., 1996; Puchta, 1998). SDSA is the default pathway for DNA damage repair in somatic cells and a higher tolerance for the alternate pathway, crossover HR could make a fundamental difference in the gene targeting efficiencies in the absence of a DSB.

Another possibility is a direct effect of repetitive DNA on gene targeting efficiencies. The 3′ terminal 400 bp of the targeting fragment contain repetitive DNA, as shown by a sequence comparison with the entire barley genome (**Figure 1Bf**). This may be true for additional areas in the 3′ terminal region since genes are absent in this part. Similar sequences occur multiple times in the genome and these may very well have an impact on gene replacement. In the absence of a DSB, a free end in the targeting fragment initiates homology search and multiple sequences with homology at other positions will compete with the target locus and thereby reduce efficiencies. The situation may be entirely different after activation of the target sequence by DSB induction since recombination occurs between preformed free ends in the genome and the targeting fragment. These repetitive sequences could not be removed since they were not detected before the barley whole genome assembly was available. But interspersed repetitive sequences are a basic problem since they cannot be removed from the gene replacement fragment without compromising homology to target locus. This problem could generally apply for gene targeting without DSB induction in all crops with complex genomes.

## Conclusion

The efficiencies of gene targeting obtained with the *ALS* locus were too low to detect one event in 4529 transformed immature embryos. This number includes experiments with P-N selection and the *PpRAD51* and *ScRAD54* overexpressing lines. Collectively, our data suggest that the natural efficiency of gene targeting in barley is too low for routine application. In addition, none of the methods tested, stimulation of gene targeting and P-N selection solved the problem. The amount of labor and time invested to transform the number of immature embryos was high and a significantly higher amount would be unreasonable. This is especially true since efficient alternatives exist. DSB induction allows high efficiency gene targeting in barley and gene replacement with reasonably sized transformation experiments was obtained with this method recently (Watanabe et al., 2016). In addition, highly efficient and easily accessible synthetic nucleases like transcription activator-like effector nucleases (TALENs) (Wood et al., 2011; Qi et al., 2013) or clustered regularly interspaced short palindromic repeats (CRISPR) and CRISPR associated protein (Cas) (CRISPR-Cas) (Fauser et al., 2014; Feng et al., 2014; Noman et al., 2016) are readily available meanwhile and make DSB induction to the method of choice in the future.

## Author Contributions

MH designed work, produced and analyzed data and drafted the manuscript. H-HS designed work and produced and analyzed data. BR designed work, produced and analyzed data and wrote the manuscript. MH, H-HS, and BR approved the manuscript and are accountable for all aspects of the work drafted.

## Conflict of Interest Statement

The authors declare that the research was conducted in the absence of any commercial or financial relationships that could be construed as a potential conflict of interest.

## Footnotes

1 http://ensembl.gramene.org/Hordeum_vulgare/Info/Index
